# Application of a Multiplex Platform to Identify Novel Biomarkers for Pregnancy Location and Viability

**DOI:** 10.21203/rs.3.rs-2777020/v1

**Published:** 2023-05-05

**Authors:** Iris Tien-Lynn Lee, Suneeta Senapati, Courtney Schreiber, Nathanael Koelper, Peter Takacs, Kurt Barnhart

**Affiliations:** University of Pennsylvania Perelman School of Medicine; University of Pennsylvania Perelman School of Medicine; University of Pennsylvania Perelman School of Medicine; University of Pennsylvania; EVMS: Eastern Virginia Medical School; University of Pennsylvania Perelman School of Medicine

**Keywords:** biomarker, ectopic pregnancy, early pregnancy loss, pregnancy viability, proteomics

## Abstract

Determining early pregnancy location and viability can be cumbersome, often requiring serial evaluations. This study aimed to identify novel biomarker candidates for pregnancy location and viability using a pseudodiscovery high through-put technique. This was a case-control study among patients presenting for early pregnancy assessment, including ectopic pregnancies, early pregnancy losses, and viable intrauterine pregnancies. For pregnancy location, ectopic pregnancy was considered “case” and non-ectopic considered “control.” For pregnancy viability, viable intrauterine pregnancy was considered “case” and early pregnancy loss + ectopic pregnancy were considered “control.” Using Proximity Extension Assay technology from Olink Proteomics, serum levels of 1012 proteins were compared separately for pregnancy location and viability. Receiver operator characteristic curves were generated to determine a biomarker’s discriminative abilities. Analysis included 13 ectopic pregnancies, 76 early pregnancy losses, and 27 viable intrauterine pregnancies. For pregnancy location, 18 markers had an area under the curve (AUC) ≥ 0.80, with three being expressed more in ectopic compared to non-ectopic pregnancies: thyrotropin subunit beta, carbonic anhydrase 3, and DEAD (Asp-Glu-Ala-Asp) box polypeptide 58. For pregnancy viability, two markers had an AUC ≥ 0.80: lutropin subunit beta and serpin B8. While some of the markers were previously identified as implicated in early pregnancy physiology, others were from pathways not previously explored. Using a high through-put platform, a large number of proteins were screened as potential biomarkers for pregnancy location and viability, and twenty candidate biomarkers were identified. Further exploration of these proteins may facilitate validation as diagnostic tools for establishing early pregnancy diagnoses.

## Introduction

In women presenting with an early pregnancy, determination of pregnancy location and viability is crucial for safety as well as expeditious management and appropriate prognostic counseling. Ectopic pregnancies (EPs) are associated with potential life-threatening complications and requires prompt and targeted treatment [1]. However, the clinician’s ability to determine the location of a pregnancy remains rudimentary, often relying on multiple visits, repeat ultrasounds, and serial serum human chorionic gonadotropin (hCG) measurements. Even once an intrauterine pregnancy (IUP) is confirmed, the clinician may not be able to tell the patient whether the pregnancy is viable until several weeks later, when follow-up ultrasound can be performed. The collective goal in treating women in early pregnancy should be to achieve the most accurate and efficient diagnosis possible.

Biomarkers – generally defined as “characteristics that are objectively measured and evaluated as indicators of normal biologic processes, pathogenic processes, or pharmacologic responses to a therapeutic intervention” – are an emerging area of interest for both pregnancy location and viability [2]. Identification of a proteomic profile sensitive for and specific to EP or EPL would allow for earlier diagnosis with the potential to avoid unnecessary interventions, save patients and clinicians time and expense, and reduce the morbidity/mortality associated with early pregnancy complications.

Prior studies have explored various potential biomarkers for pregnancy location and viability, with a number of candidate biomarkers often used in combination to achieve maximal accuracy. However, despite utilization of such panels of markers, there is poor reproducibility and external validation in this application of proteomics [3]. The question of whether other previously unexplored biomarkers could facilitate these efforts remains unanswered.

Olink Proteomics utilizes proprietary dual-recognition, DNA-coupled Proximity Extension Assay (PEA) technology to perform high throughput analyses of over a thousand highly validated protein targets [4]. While traditional proteomic discovery studies utilize mass spectrometry, application of the PEA platform for pseudodiscovery offers a more efficient, complementary alternative. Using this platform, we aimed to identify novel biomarker candidates for pregnancy location and viability and to characterize comparative proteomes of three pregnancy outcomes – EP, EPL, and vIUP.

## Materials and methods

### Study design

We conducted a case-control study among patients presenting for early pregnancy assessment, including EPs, EPLs, and vIUPs. Information on demographic characteristics were collected at the time of enrollment, and determination of pregnancy outcome was achieved by chart review. All outcomes were determined during the course of routine clinical care. Final pregnancy outcomes were designated using the following criteria:

1. EP – visualization of an extrauterine gestation during surgery, ultrasound demonstrating an adnexal mass without evidence of an intrauterine pregnancy, or an increase in hCG level after uterine evacuation
2. EPL – embryonic loss (fetal pole > 4 mm with no cardiac activity) or an anembryonic gestation (gestational sac > 16 mm with no identified yolk sac or fetal pole) or with no change in size of fetal pole or gestational sac one week apart with evidence of products of conception on histopathology
3. vIUP – intrauterine gestational sac, yolk sac, and fetal pole with cardiac activity
  - Early vIUP – less than 8 weeks gestational age based on ultrasound dating
  - Late vIUP – more than 8 weeks gestational age based on ultrasound dating

For the question of pregnancy location, EP was considered “case” and non-EP (EPL + early vIUP) considered “control.” Only early vIUPs were included in the non-EP category because the gestational ages were more comparable to those seen in EPs and EPLs. For the question of pregnancy viability, vIUP (including early and late vIUPs) was considered “case” and EPL + EP was considered the nonviable “control.”

Inclusion criteria were age 18 years or older and a confirmed pregnancy outcome. Exclusion criteria included prior treatment for the current pregnancy, gestational trophoblastic disease, and possible multiple gestation. In order to obtain the study cohort, all available samples that were eligible were included. The study was approved by the institution’s Internal Review Board.

### Data collection and biomarker assays

Serum was collected at the point of initial presentation, centrifuged at 1,500 rpm for 5 minutes, split into 0.5-mL aliquots, and stored at −80C. Serum samples from participants were analyzed by Olink Proteomics 96-plex protein panel kits including a total of 1012 proteins. We selected all 11 panels provided by Olink in order to maximize our discovery capability; the analyzed proteins included previously identified biomarkers with physiologic basis in early pregnancy as well as previously unexplored biomarkers without known association with early pregnancy physiology.

Olink applies PEA technology, using pairs of antibodies linked to oligonucleotides (PEA probes) with affinity to one another [5]. Once the target binds the probes, they are brought together and then extended by a DNA polymerase that creates a new surrogate marker for the target antigen. Quantitative real-time PCR (qPCR) is then used to quantify this marker. Olink allows for simultaneous detection of multiple biomarkers, enabling creation of a biomarker “signature” for a given characteristic or condition. Protein concentrations are reported in Normalized Protein eXpression (NPX), an arbitrary unit in log2 scale used by Olink to minimize both intra- and inter-assay variation.

### Quality control

Olink performs standard internal quality control (QC) analyses on all samples. Four internal controls are added to each submitted sample to monitor the quality of assay performance. This is then expressed as normalized protein expression (NPX), which is Olink’s arbitrary unit in log2 scale. The quality of each sample is then determined by evaluating the deviation from the median value of the controls for each individual sample, and those with a deviation of less than 0.3 NPX are considered to pass QC. Samples with an intra-assay coefficient of variation (CV) of greater than 15% or an inter-assay CV of greater than 20% were excluded.

### Statistical analysis

For demographic and clinical characteristics, Kruskal-Wallis test was used for continuous variables, and Pearson Chi-square or Fisher-exact test was used for categorical variables.

Serum protein concentrations of all 1012 proteins were compared between cases and controls using two-sided *t*-tests, with separate comparisons performed for pregnancy location (EP versus EPL and early vIUP) and viability (EPL and EP versus vIUP). The discriminatory ability of a given biomarker to differentiate between pregnancy outcomes was determined by calculation of an area under receiver operating characteristic curve (AUC). An AUC of 0.5 was considered to be consistent with chance, while an AUC of 0.8 or higher was considered to be highly predictive. On the converse, for biomarkers with a negative association with the outcome (i.e. low protein levels in EP or EPL compared to controls), an AUC of 0.2 or below was considered highly predictive; these data are presented as 1-AUC for consistency of interpretation with the other biomarkers. Adjustment was made for the differences in gestational age among phenotypes. To correct for multiple comparisons, the Benjamini-Hochberg procedure was applied with corrected *p*-value thresholds of 0.003 for pregnancy location and 0.004 for pregnancy viability.

## Results

### Demographics and clinical characteristics

The study sample consisted of 128 participants: 16 EPs, 80 EPLs, and 32 vIUPs (16 early, 16 late). After excluding samples dropped following quality control, the final sample included 13 EPs, 76 EPLs, and 27 vIUPs (13 early, 14 late), resulting in a sample size of 116 participants. Demographic and clinical characteristics of the participants are shown in Table 1. Twelve participants were dropped because their samples did not pass QC (Supplemental Table 1).

### Biomarkers predicting pregnancy location

**Figure 1:**
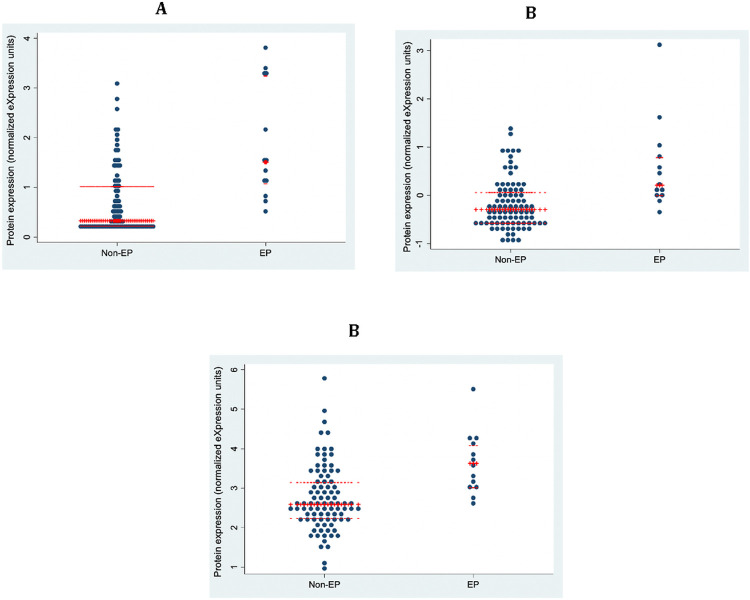
Biomarkers with higher expression in ectopic compared to non-ectopic pregnancies. (a) TSHB, (b) CA3, and (c) DDX58 have higher expression in ectopic compared to non-ectopic pregnancies, with the difference most pronounced in TSHB. Converted from log2 NPX to linear scale, the mean level of TSHB was 2.27 times higher in ectopic versus non-ectopic pregnancies.

Using an AUC threshold of ≥ 0.75, 60 markers predicting pregnancy location were identified, while eighteen markers were highly predictive with an AUC ≥ 0.80 (Table 2). Most of these markers had lower levels of expression in EP compared to EPL + early vIUP, but three markers were higher in EP: thyrotropin subunit beta (TSHB), carbonic anhydrase 3 (CA3), and DEAD (Asp-Glu-Ala-Asp) box polypeptide 58 (DDX58). Converted from log2 NPX to linear scale, the mean level of TSHB in EP was 2.27 times that seen in the comparison group. Similarly, CA3 expression was 1.71 times higher in cases compared to controls, and DDX58 expression was 1.85 times higher. (Figure 1) The functions of other highly predictive biomarkers are described in Supplemental Table 2.

### Biomarkers predicting pregnancy viability

**Figure 2:**
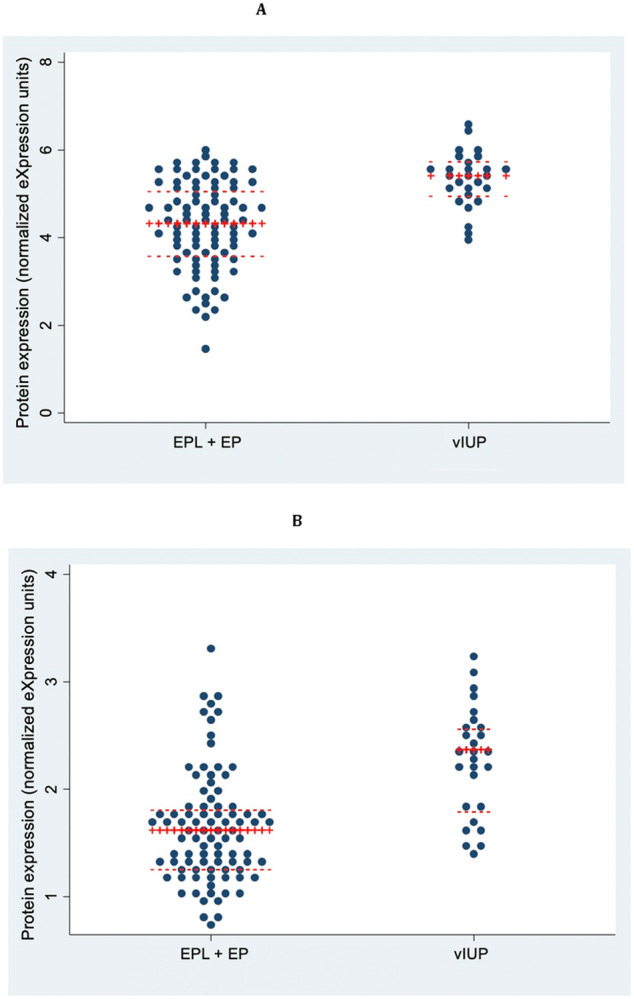
Biomarkers with higher expression in viable compared to non-viable pregnancies. (a) LHB and (c) SERPINB8 have higher expression in viable compared to non-viable pregnancies.

Ten biomarkers were identified with an AUC of ≥ 0.75 for pregnancy viability. Two of these markers had an AUC ≥ 0.80 (Table 3): lutropin subunit beta (LHB) and serpin B8 (SERPINB8). Both had higher expression in vIUPs compared to EPL + EP (Figure 2).

## Discussion

Using a pseudodiscovery high through-put platform, we aimed to screen a large number of proteins as potential biomarkers for pregnancy location and viability. We were able to identify multiple markers to help differentiate between pregnancy outcomes: eighteen markers were highly predictive of pregnancy location while two were highly predictive of pregnancy viability.

In most of the markers that were highly predictive in the pregnancy location comparison, expression was lower in EP versus non-EP (EPL + early vIUP). However, there were three proteins that had higher levels of expression in EP. These proteins have the potential to serve as clinical biomarkers to determine whether or not a pregnancy is ectopic. Though there is biological plausibility for many of these biomarkers and some have been explored previously, some also arise from pathways that are less clearly related to early pregnancy physiology.

The biomarkers that were higher in EP compared to non-EP were TSHB, CA3, and DDX58. TSHB is the unique beta-subunit in thyroid stimulating hormone (TSH) [6]. In general, thyroid hormone is thought to play a role in modulating feto-maternal tolerance and angiogenesis during implantation [7]. CA3 is a zinc metalloenyzme that catalyzes the reversible conversion of carbon dioxide to carbonic acid [8]. As a group, carbonic anhydrases are thought to modulate uterine morphogenesis and endometrial gland development in various non-human mammals [8, 9]. Furthermore, mouse studies suggest that carbonic anhydrases serve as negative regulators in implantation, development, and maintenance of pregnancy [10]. DDX58 is an RNA helicase involved in viral double-stranded RNA recognition and the regulation of the antiviral innate immune response [11]. Studies in pregnant heifers have demonstrated that DDX58 is present in the endometrium of early pregnancy and is regulated by the conceptus [12]. It remains unclear if these biomarkers play a direct role in early pregnancy physiology, and if so, the mechanism by which they affect location of implantation is unknown.

The most predictive biomarkers for pregnancy location were pregnancy-associated plasma protein-A (PAPP-A) and tissue factor pathway inhibitor 2 (TFPI-2), both lower in EP compared to non-EP. PAPP-A is a metalloproteinase that cleaves insulin-like growth factor binding protein 4 (IGFBP-4), resulting in release of bound IGF [13]. Its predictive value for EP has been previously reported, and our findings corroborate the lower levels of PAPP-A seen in EP [14, 15]. TFPI-2 is a proteinase inhibitor produced by the placenta. Prior studies have identified a possible association between TFPI-2 levels and preeclampsia, suggesting that it may play an inhibitory role in modulating the invasiveness of trophoblast cells during implantation [16]. While these biomarkers are lower in EP, they may help to shed light on underlying early pregnancy physiology and pathology.

For pregnancy viability, levels of both markers with high predictive value were higher in viable compared to nonviable pregnancies. Previous studies looking at expression of *LHB* in pregnant mares found that *LHB* is upregulated compared to in nonpregnant mares, and it subsequently increases release of LH and chorionic gonadotropin (CG) into the uterine environment [17]. SERPINB8 is a protease inhibitor with unclear significance in early pregnancy, though a prior study found that the cumulus cells of oocytes resulting in a pregnancy expressed much higher levels of SERPINB8 compared to the oocytes that did not result in a pregnancy [18]. Our work examined serum levels rather than local levels in the oocytes so it is difficult to compare findings, but further exploration of the relevance of SERPINB8 in pregnancy viability is warranted.

A key strength of this study is the inclusion of a large number of diverse proteins as potential biomarkers. An agnostic approach to the identification of new biomarkers is important, as demonstrated by the findings that the proteins with highest predictive value were not all in pathways that are well recognized in reproductive physiology. Identification of these new biomarkers may also advance our understanding of early pregnancy physiology by drawing attention to previously unexplored pathways. Another strength of this study is that the pregnancy outcomes were either sonographically or histologically confirmed, making it possible to draw accurate conclusions about the proteomics of the different cohorts.

An important limitation of this study is the possibility of false discovery given the large number of proteins screened. However, the Benjamini-Hochberg correction was applied to account for multiple comparisons, and results remained statistically significant. Additionally, the difference in hCG levels among the phenotypes was not adjusted for as a confounder, as it reflects biological differences inherent to the different pregnancy outcomes. Another limitation is that the sample size, particularly for EPs, was small. However, this study aimed to provide preliminary evidence to guide further biomarker research, and we were able to establish high discriminatory ability even with the small number of EPs. Despite these limitations, the findings represent a first step toward identifying which biomarkers to focus on, and future work should validate these findings using prospective screening in order to establish their clinical application.

In identifying multiple highly predictive biomarkers for both pregnancy location and viability, this study provides a foundation for understanding the possible role of proteomics to diagnosis and management of early pregnancy. While clinical application remains many steps away, the ultimate use of biomarkers to inform clinical decision making has the potential to be of great utility in risk stratification for pregnancy of unknown location as well as management and counseling of nonviable pregnancies.

## Figures and Tables

**Table 1.** Demographic and clinical characteristics

-	EP	EPL	vIUP	*p*- value
Age	32.4 (5.8)	29.7 (6.9)	28.7 (6.3)	0.34
Race				0.22
White	4 (30.8)	45 (37.5)	6 (22.2)	
Black	7 (53.9)	54 (45.0)	19 (70.4)	
Other	2 (15.4)	21 (17.5)	2 (7.4)	
Initial HCG (mIU/mL)	6126 (2329-6433)	27312 (11900-50592)	68626 (47390-77981)	<0.001
Gestational age at presentation by last menstrual period	37.4 (14.7)	59.1 (12.1)	46.7 (14.5)	<0.001
Gravidity	3 (2-3)	3 (2-5)	2 (14)	0.66
Parity	1 (0-2)	1 (0-3)	1 (0-2)	0.92

Data are presented as mean (standard deviation) for parametrically distributed variables and median (interquartile range) for non-parametrically distributed variables

**Table 2.** Pregnancy Location Markers with AUC ≥ 0.8

Pregnancy Location (EP versus EPL + early vIUP)					
Marker	Mean (Standard Deviation)	Mean (Standard Deviation)	Area Under the Curve (95% CI)	Adjusted** Area Under the Curve (95% CI)	*p*-value***
	EP	EPL + early vIUP			
PAPP-A*	3.67 (1.92)	7.06 (1.04)	0.92 (0.87-0.98)	0.95 (0.90-1.00)	<0.001
TFPI2*	8.94 (0.57)	11.36 (1.53)	0.91 (0.85-0.97)	0.92 (0.86-0.98)	<0.001
SIGLEC6*	4.83 (0.39)	6.26 (1.08)	0.90 (0.84-0.96)	0.95 (0.91-1.00)	<0.001
DDR1*	6.35 (0.17)	6.70 (0.23)	0.90 (0.83-0.96)	0.95 (0.90-1.00)	<0.001
LYPD3*	6.78 (0.32)	6.37 (0.47)	0.89 (0.80-0.98)	0.86 (0.76-0.96)	<0.001
ANGPT2*	2.50 (0.41)	3.98 (1.15)	0.88 (0.81-0.95)	0.93 (0.88-0.98)	<0.001
GDF_15*	4.94 (0.74)	6.49 (1.22)	0.87 (0.80-0.94)	0.88 (0.80-0.97)	<0.001
TLT2*	4.72 (0.44)	5.44 (0.50)	0.87 (0.77-0.97)	0.92 (0.80-1.00)	<0.001
TSHB	1.89 (1.17)	0.71 (0.68)	0.84 (0.75-0.93)	0.87 (0.77-0.97)	<0.001
IL27*	4.82 (4.55)	5.55 (0.56)	0.84 (0.75-0.94)	0.93 (0.85-1.00)	<0.001
CGA*	9.36 (0.93)	10.24 (0.98)	0.83 (0.74-0.92)	0.86 (0.73-0.98)	<0.001
LIFR*	3.22 (0.16)	3.64 (0.45)	0.83 (0.74-0.92)	0.91 (0.84-0.97)	<0.001
CCL19*	8.45 (0.62)	9.34 (0.86)	0.81 (0.69-0.93)	0.90 (0.83-0.98)	<0.001
OPN*	3.39 (0.57)	4.05 (0.50)	0.81 (0.67-0.96)	0.93 (0.87-0.98)	<0.001
CA3	0.58 (0.94)	−0.19 (0.51)	0.81 (0.71-0.91)	0.89 (0.80-0.99)	<0.001
DDX58	3.64 (0.78)	2.75 (0.83)	0.81 (0.71-0.91)	0.89 (0.79-0.99)	<0.001
ENRAGE*	5.38 (0.68)	6.29 (0.99)	0.81 (0.71-0.90)	0.89 (0.80-0.98)	0.002
CHRDL2* 2.93 (0.53)	3.62 (0.57)	0.80 (0.69-0.92)	0.89 (0.78-1.00)	<0.001

* Denotes negative association between biomarker and outcome (lower levels of biomarker seen in EP compared to EPL + early VIUP); for these proteins, 1-AUC is reported in this table for consistency

** Adjusted for gestational age

*** Corrected *p*-value after Benjamini-Hochberg adjustment for multiple comparisons

Data are reported in Normalized Protein eXpression (NPX) units, which are in log2 scale

**Table 3.** Pregnancy Viability Markers with AUC ≥ 0.8

Pregnancy viability (early + late vIUP versus EPL + EP)					
Marker	Mean (Standard Deviation)	Mean (Standard Deviation)	Area Under the Curve (95% CI)	Adjusted* Area Under the Curve (95% CI)	*p*-value**
	Early + late vIUP	EPL + EP			
LHB	5.33 (0.64)	4.28 (1.00)	0.80 (0.72-0.89)	0.81 (0.71-0.91)	<0.001
SERPINB8	2.26 (2.06)	1.64 (0.52)	0.80 (0.71-0.89)	0.82 (0.71-0.93)	<0.001

* Adjusted for gestational age

** Corrected *p*-value after Benjamini-Hochberg adjustment for multiple comparisons

Data are reported in Normalized Protein eXpression (NPX) units, which are in log2 scale

## Data Availability

Data will be made available upon reasonable request.

## Code availability:

Code will be made available upon reasonable request
